# Positive contrast spiral imaging of a nitinol guidewire

**DOI:** 10.1186/1532-429X-17-S1-Q15

**Published:** 2015-02-03

**Authors:** Adrienne E Campbell-Washburn, Toby Rogers, Burcu Basar, Merdim Sonmez, Ozgur Kocaturk, Robert J Lederman, Michael Hansen, Anthony Z Faranesh

**Affiliations:** 1 Division of Intramural Research, Cardiovascular and Pulmonary Branch, National Heart Lung and Blood Institute, National Institutes of Health, Bethesda, MD, USA; 2 Institute of Biomedical Engineering, Bogazici University, Istanbul, Turkey

## Background

The clinical translation of MRI-guided cardiovascular catheterization has been limited by the unavailability of devices that are both visible and safe under MRI. In particular, rigid metallic guidewires are essential for most catheterization procedures and are at risk of heating during MR imaging [1]. Here we present an MRI method that simultaneously improves the visualization of commercially available nitinol guidewires and minimizes RF induced heating.

## Methods

RF-efficient gradient echo spiral imaging was chosen to minimize heating (8 interleaves, TE/TR = 0.86/10ms, flip angle = 10°). Through-slice dephasing generated a positive contrast "device image" [2], exploiting local field inhomogeneity such that the metallic guidewire appears hyperintense with background signal suppressed. An anatomical image and a device image were interleaved in alternating frames. Image processing (signal thresholding and selection of elongated structures) was performed on the device image to isolate the guidewire signal from other sources of field inhomogeneity.

Imaging was performed on a 1.5T MRI scanner (Aera, Siemens, Erlangen, Germany). MRI-guided left heart catheterization was performed in a pig using a 0.035" commercially available nitinol guidewire (Nitrex, Covidien, Plymouth, MN). The RF induced temperature rise at the tip of an insulated nitinol rod during MR imaging was measured in the ASTM 2182 gel phantom using a fiberoptic temperature probe (OpSense, Quebec, Canada).

## Results

**Figure 1:**
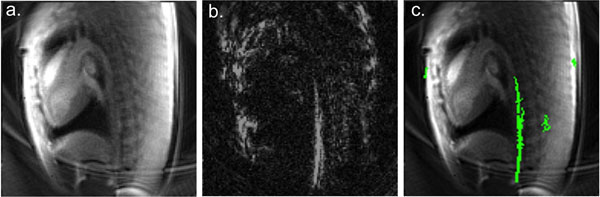
An example frame from MRI-guided left-heart catheterization in a pig using spiral imaging for interleaved anatomical images (a) and positive contrast device images (b). Real-time image processing was used to produce a color overlay of the guidewire on the anatomy for procedural guidance (c).

A pair of anatomical and device images were generated with a temporal resolution of 160ms (80ms per image), or 6.25 frames/s. Image processing occurred in real-time and a color overlay of the device on the anatomy was displayed to the operator for guidance during catheterization (Figure 1). Isolation of the guidewire signal from the background was most challenging around the aortic arch.

**Figure 2:**
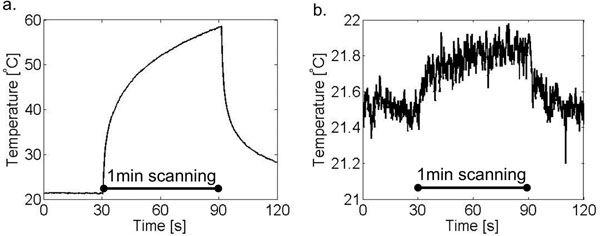
In vitro temperature measurement at the tip of an insulated nitinol rod. Cartesian imaging (flip angle = 45°, TE/TR =1.27/2.54 ms) generated 37.2°C of heating (a) and spiral imaging (flip angle = 10°, TE/TR = 0.86/10 ms) generated 0.47°C of heating (b) during 1 minute of continuous scanning.

During 1 minute of continuous scanning in vitro, our spiral imaging method generated 0.47°C at the tip of a nitinol rod, compared to 37.2°C of heating generated using standard Cartesian bSSFP imaging (Figure 2).

## Conclusions

This method simultaneously improves guidewire visualization using a real-time color overlay and minimizes heating using RF-efficient imaging. Thus, this method may permit the safe and effective use of standard commercially available metallic guidewires for MRI-guided cardiovascular catheterizations.

## Funding

This work was supported by the NHLBI DIR (Z01-HL006039-01, Z01-HL005062-08).
